# Biomimetic Hydrogels for In Vitro Modelling of Nucleus Pulposus Degeneration: Effects of Extracellular Matrix Compositional Change on Physicochemical Properties and Cell Phenotype

**DOI:** 10.3390/jfb16070253

**Published:** 2025-07-08

**Authors:** Narjes Rashidi, Nicholas Dowell, Derek Covill, John Shepperd, Matteo Santin

**Affiliations:** 1 Centre for Regenerative Medicine and Devices, University of Brighton, Huxley Building Lewes Road, Brighton BN2 4GJ, UK; narjes_rashidi@yahoo.com (N.R.); littlemint899@gmail.com (J.S.); 2 School of Applied Sciences, University of Brighton, Huxley Building Lewes Road, Brighton BN2 4GJ, UK; 3 Clinical Imaging Sciences Centre, Brighton and Sussex Medical School, BSMS Teaching Building, University of Sussex, Brighton BN1 9PX, UK; 4 School of Architecture Technology and Engineering, University of Brighton, Advanced Engineering Building Lewes Road, Brighton BN2 4GJ, UK

**Keywords:** intervertebral disc degeneration, nucleus pulposus ECM changes, nucleus pulposus-mimicking hydrogels, nucleus pulposus collagen–proteoglycan hydrogel, in vitro model for degenerated nucleus pulposus

## Abstract

The intervertebral disc, an anatomical compartment interposed between vertebral bodies, plays a key role in spine flexibility and compression loading. It comprises three tissues: the nucleus pulposus, the annulus fibrosus, and the end plates. Degeneration-related changes in the extracellular matrix of the nucleus pulposus upon ageing or pathological conditions prompted the present investigation into the impact of proteoglycan reduction, the main constituent of the healthy nucleus pulposus, on its physicochemical properties and cellular phenotypical changes. To mimic the native extracellular matrix, three-dimensional NP-mimicking constructs were developed using a biomimetic hydrogel composed of collagen type I, collagen type II, and proteoglycans. This system was fabricated using a bottom-up approach, employing highly pure monomeric collagen types I and II, which were induced to form a reconstituted fibrillar structure closely resembling the natural NP microenvironment. A comprehensive physicochemical characterization was conducted at varying proteoglycan percentages using scanning electron microscopy (SEM), FTIR, rheological tests, and water retention property analysis. The effect of microenvironment changes on the phenotype of nucleus pulposus cells was studied by their encapsulation within the various collagen–proteoglycan hydrogels. The morphological and immunochemistry analysis of the cells was performed to study the cell–matrix adhesion pathways and the expression of the cellular regulator hypoxia-inducible factor 1 alpha. These were linked to the analysis of the synthesis of healthy or pathological extracellular matrix components. The findings reveal that the reduction in proteoglycan content in the nucleus pulposus tissue triggers a pathological pathway, impairing the rheological and water retention properties. Consequently, the cell phenotypes are altered, inducing the synthesis of collagen type I rather than securing the natural physiological remodelling process by the synthesis of collagen type II and proteoglycans. Identifying the proteoglycan content threshold that triggers these pathological phenotypical changes could provide new diagnostic markers and early therapeutic strategies for intervertebral disc degeneration.

## 1. Introduction

The intervertebral disc (IVD) is a cartilaginous tissue that shares biochemical similarities with articular cartilage but possesses distinct morphological characteristics [1]. The IVD is composed of three key compartments: the nucleus pulposus (NP), the annulus fibrosus (AF), and the end plates. The NP, a highly hydrated and plastic region, is surrounded by the AF, which consists of a network of collagen fibres arranged in sheets encircling the nucleus [2]. This fibrous structure not only imparts tensile strength but also restricts distortion of the anatomy under load, containing the pressure of the NP and maintaining it within its boundaries [3]. If the load or distortion exceeds the structure’s mechanical strength, damage occurs, triggering a repair mechanism.

The AF and the NP, in conjunction with the facet joints, allow compliance of the axial skeleton. By limiting motion at the level of the individual vertebral segments, the disc avoids damage to the adjacent neural elements. The sum of these segments enables the required function while resisting tensile and torsional forces [4,5]. The unique vertical posture of humans makes them susceptible to signs of damage repair. Most observable changes are likely the result of repair processes. Several factors—including ageing, trauma, genetics, and excessive mechanical loading—likely contribute to the onset of IVD degeneration, which significantly impairs both structure and function [6]. The IVD undergoes identifiable ageing and degenerative alterations at earlier stages of life compared to other connective tissues of the body [7]. During this degeneration process, significant changes occur in both the cellular and extracellular matrix (ECM) composition [8]. A decrease in cell density, together with the transition of NP cells from large notochordal cells towards chondrocyte-like cells (i.e., smaller cell clusters nested in lacunae) [9], results in the synthesis of a non-specific NP matrix [10]. Hence, the NP undergoes a fibrous transformation, from transparent hyaline cartilage to denser, opaque fibro-cartilaginous tissue (marked by a shift from predominantly collagen type II synthesis to collagen type I) [8,11]. These changes in ECM composition lead to a loss of NP mechanical and cellular activity, affecting metabolism, proliferation, differentiation, and survival [10].

The NP ECM is composed of a network of different proteins, mainly consisting of collagen type II, proteoglycans (PGs), and water [12]. The ECM provides mechanical support, retaining disc hydration and elasticity, and it facilitates nutrient delivery to cells and the removal of catabolites through cycles of compression and relaxation, respectively [13]. During disc degeneration, the balance between anabolic and catabolic processes is disrupted, leading to the progressive degradation of ECM components. This degradation is driven by the overexpression of matrix metalloproteinases (MMPs) and ADAMTS (a disintegrin and metalloprotease with thrombospondin motifs) [14,15,16]. This leads to dehydration and progressive ECM disorganization, which promotes mechanical failure, annular tears, and other characteristic features of disc degeneration [3]. In addition to structural changes—such as the replacement of collagen type II with type I, reduced aggrecan synthesis, and disruption of long proteoglycan chains, which may contribute to nociceptive signalling—inflammatory mediators like cytokines may also be released. These factors further impair cell activity and tissue homeostasis [17]. This imbalance in the normal homeostatic mechanism impairs normal disc function and disc cell metabolism, particularly in the NP, and may contribute to herniation, nerve compression, and spinal pain [18].

Another major change in the ECM during degeneration is the loss of proteoglycans, which are large molecules with a high capacity for water retention. The decrease in PG content results in disc dehydration and reduced disc height [19,20]. In addition, the loss of PGs impairs the disc’s mechanical properties—particularly compressive stiffness—thereby increasing the risk of damage under compressive forces [21].

Understanding the mechanisms that regulate cell–ECM interactions is likely crucial for comprehending how ECM changes influence cell survival, matrix synthesis, and metabolism. Integrins, a class of cell surface receptor molecules, play a key role in mediating cell–ECM interactions, including cell adhesion and migration [22,23]. Furthermore, integrin–ECM interactions can modulate cell signalling, thereby impacting important cellular functions such as cell survival, proliferation, and protein production [24]. Integrins are heterodimers composed of α and β subunits that work together to interact with a variety of ligands. In the context of IVD, the α5 and β1 integrin subunits are known to facilitate interactions with collagens and fibronectin [25]. Previous studies have reported elevated levels of α5 and β1 integrin subunits in herniated IVD tissue, suggesting their involvement in pathology [26,27,28]. On the other hand, CD44 signalling has been associated with the modulation of inflammatory responses. CD44, a cell adhesion molecule expressed on chondrocytes, mediates adhesion to extracellular matrix glycosaminoglycans and hyaluronan [29]. Additionally, hypoxia-inducible factor 1α (HIF-1α) has emerged as a significant biomarker implicated in IVD degeneration, potentially affecting multiple pathways [30].

The present in vitro study aimed to investigate possible links between the age-related changes in the physicochemical properties of the main NP extracellular matrix (ECM) components and the alterations in cell behaviour that potentially lead to pathological conditions. Accordingly, the present work investigated the influence of PG content on the expression of HIF-1α, as the interplay between PGs and HIF in NP cells has not been documented before. In addition, the study explored cell adhesion pathways mediated by integrin-β1 and CD44 receptors. We believe that this signalling pathway could potentially contribute to the degeneration process of IVD.

Various biopolymers have been used to produce intervertebral disc (IVD) constructs [31]. Among them, collagen type II hydrogels have been shown to increase cell viability without affecting the nucleus pulposus (NP) phenotype [32]. Similarly, the microencapsulation of NP cells in a 3D microsphere system made of collagen type I maintained a round NP cell morphology while preserving NP phenotypic markers, such as type II collagen and cytokeratin-19 [33]. Additionally, collagen type II cross-linked with genipin has been used to promote the differentiation of adipose-derived stem cells (ASDCs) into NP-like cells via the Shh signalling pathway [34]. Genipin has also been used to stabilize collagen type II and the chondroitin sulfate gel, enhancing NP-like expression in ASDCs and enabling the partial restoration of NP tissue [35]. Building upon these approaches, our research focuses on a hydrogel that combines collagen type I, collagen type II, and proteoglycans to develop a biomimetic in vitro ECM model of the nucleus pulposus. This model more closely mimics the native intervertebral disc ECM compared to previous studies. Furthermore, we employed a bottom-up approach for hydrogel fabrication, using highly pure monomeric collagen type I and type II, which were induced to form a reconstituted fibrillar structure. To the best of our knowledge, this is the first instance of using atelocollagen/monomeric collagens to create an ECM model for IVD degeneration. While atelocollagen has been used in IVD tissue engineering and other applications like bone and cartilage repair [36,37,38], its use in creating a degenerative ECM model is novel. In this study, proteoglycans isolated from bovine nasal cartilage were selected due to their availability, compositional consistency, and structural similarity to native aggrecan. While differences in glycosaminoglycan chain length and sulfation patterns exist between nasal cartilage and nucleus pulposus-derived aggrecan, the former was deemed adequate to retain the key hydrophilic and structural features essential for mimicking native ECM in early-phase construct/hydrogel development.

## 2. Materials and Methods

### 2.1. Fabrication of Collagen and Proteoglycan Hydrogels and Cell Encapsulation

Three-dimensional (3D) hydrogel constructs were developed to closely mimic either the physiological or pathological conditions of the nucleus pulposus (NP). Based on data published in the literature, the constructs combined collagen type II (75%) and collagen type I (25%) with varying concentrations of proteoglycans (0%, 30%, and 70% *w*/*w*) [39]. Collagen monomers were induced to form reconstituted fibrillar structures under certain conditions (i.e., buffer, pH, and temperature) as described hereinafter. To prepare 1 mL of a collagen–PG hydrogel with the aforementioned ratios of the components, 750 µL of collagen type II solution (Koken, Kakegawa-City, Japan) was mixed with 150 µL of collagen type I solution (Collagen Solutions company, Eden Prairie, MN, USA, bovine skin) in a microcentrifuge tube. To stimulate collagen self-assembly/fibrillogenesis, 100 µL of 10× phosphate-buffered saline (PBS) (Merck, London, UK) was then added to the afibrillar collagen solutions and mixed well. To raise the pH to a neutral pH, 3 µL of NaOH (1M) was added to the mixture. Proteoglycans (extracted from bovine nasal cartilage, Sigma Aldrich, Gillingham, UK) were subsequently added to the solution and mixed thoroughly. The nucleus pulposus cell suspension was mixed with the collagen hydrogel, and 100 µL of hydrogel-encapsulated cells (seeding density of 10^5^ per 100 µL of the gel) was transferred to a 96-well plate (Falcon Corning, Glendale, AZ, USA) and incubated at 37 °C under static conditions.

### 2.2. Rheological Analysis

The viscoelastic properties of the collagen preparations, including storage modulus (G’), loss modulus, and complex viscosity (η*), were obtained using a HAAKE Mars rheometer. An oscillatory frequency sweep test was conducted within the linear viscoelastic region (LVR) at 1% strain with an angular frequency of 0.2–2% and a temperature of 20 °C. A cone-on-plate system (30 mm diameter) was used with a gap size of 0.035 mm.

### 2.3. Fourier-Transform Infrared (FTIR) Spectroscopy

FTIR spectroscopy was used to confirm chemical interactions and/or bond formation between PGs and type II collagen by the variation in the PG content of the gels. Spectra were obtained by Perkin Elmer Spectrum Two, and samples were analysed in the transmittance mode at a 4 cm^−1^ resolution, 64 times over the range of 500–4000 cm^−1^.

### 2.4. Microstructural Analysis by Cryo-Scanning Electron Microscopy (Cryo-SEM)

A Cryo-SEM, using a Quorum Technologies PP3000T with a cryogenic sample preparation system and Zeiss SIGMA FEG-SEM, was operated at 5 kV. Specimens were mounted on SEM stubs, rapidly frozen in liquid nitrogen, placed under vacuum conditions, knife-fractured, coated with 4 nm of platinum under the same conditions, and finally transferred into an SEM chamber.

### 2.5. Water Retention Capacity Test

Samples of the different hydrogel formulations (200 µL volumes) were transferred to small cylindrical glasses (height: 30.5 mm; diameter: 4.8 mm; thickness: 0.7 mm) and incubated at 37 °C for 30 min until gelified. Samples’ water loss was measured over a period of 96 h at different time points.

### 2.6. Cellular Characterization

#### 2.6.1. Cell Culture

Human primary NP cells were purchased from ScienCell Co. (Carlsbad, CA, USA). Cells were cultured and grown in a T75 flask (Nunclon, Sarstedt, Leicester, UK) according to the supplier’s instructions. Briefly, a T75 flask was coated with Poly-L-Lysine (PLL), and cells were transferred to the flask and fed with an NP cell medium (NP cell growth supplement (1% *v*/*v*), foetal bovine serum (2%/*v*/*v*), and penicillin/streptomycin solution (1% *w*/*v*, ScienCell, US)) every 2–3 days. They were sub-cultured once 80% confluency was reached and used in passage 4 for the study.

#### 2.6.2. Image Analysis

Cell viability: Cell viability in hydrogels was examined by Live/Dead assays following the manufacturer’s instructions (Molecular ProbesTM, Invitrogen, Inchinnan, UK) and was imaged using a Leica TCS SP5 confocal laser scanning microscope (Leica Microsystems, Heidelberg, Germany).

Immunohistochemistry: The hydrogel-encapsulated cells were fixed with paraformaldehyde (PFA, Sigma Aldrich, UK) and washed twice with phosphate-buffered saline (PBS, Sigma Aldrich, UK). They were then incubated with 1% (*w*/*v*) bovine serum albumin (BSA, Sigma Aldrich, UK) for 1 h at room temperature. They were subsequently incubated overnight at 4 °C with antibodies (primary monoclonal anti-mouse anti-human), including primary integrin beta 1, CD44, and HIF-1a (1:100, Abcam, Cambridge, UK). Samples were later incubated with either 488 or 594 nm fluorophore-conjugated secondary antibodies (1:100, Fisher Scientific, Loughborough, UK) for 1 hr at room temperature in the dark and counterstained with DAPI (4′,6-diamidino-2-phenylindole, Fisher Scientific, Loughborough, UK). The antibodies’ specific reactivity with biomarkers was validated by staining acellular constructs. Images were taken using a confocal microscope (Leica TCS SP5) with ×10 and ×20 objective lenses.

Histological analysis: PFA-fixed samples were stained with Pico-Sirius Red and Alcian Blue (Sigma Aldrich, UK) by standard protocols to visualize collagen and sulfated glycosaminoglycans, respectively. Images were taken by light microscopy (Nikon Eclipse E200, Tokyo, Japan) at ×10 magnification.

### 2.7. Statistical Analysis

All quantitative data are presented as mean ± standard deviation. For rheological, FTIR, and water loss tests, the sample size was *n* = 3. For cell-based assays, *n* = 3 biological replicates were used. Statistical analyses for initial water content and water loss measurements were conducted using a one-way or two-way analysis of variance (ANOVA) in GraphPad Prism v9.3.1. A significance level of *p* < 0.05 was applied. Quantitative analysis of fibril diameters was performed using ImageJ 1.51. Fibril diameters are reported as mean ± standard deviation (SD), with n = 15 fibrils per group. A priori power analysis was not performed due to the exploratory nature of the study.

## 3. Results

### 3.1. Rheological Analysis

Hydrogels’ rheological behaviour can help clarify the influence of the introduction of PGs into the collagen suspension. Rheological properties, together with the chemical bonding (FTIR), help explain the hydrogel microstructure and the consequent cellular response.

**Figure 1:**
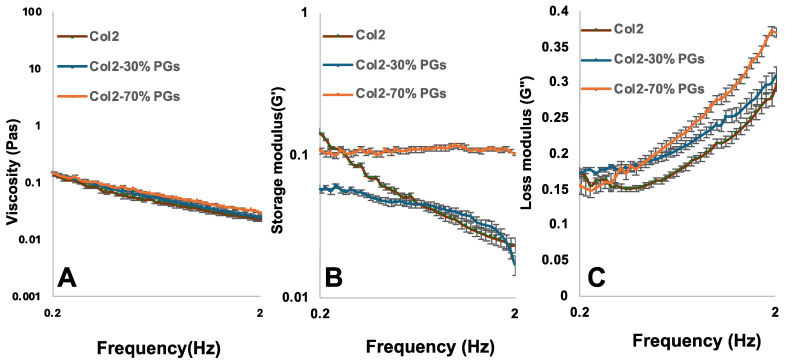
Effect of PG concentration change on the rheological properties of mimicking NP constructs. (**A**) Complex viscosity, (**B**) storage modulus, and (**C**) modulus loss of collagen II, collagen II–PGs 30% *w*/*w*, and collagen II–PGSs 70% *w*/*w* in an oscillatory shear test; frequency sweep: angular frequency = 0.2–2 Hz; strain amplitude γ = 1%, T = 20 °C.

The material response to increasing frequency was examined at a constant strain and temperature. Dynamic frequency sweeps were conducted within the linear viscoelastic region (LVR) at 1% strain with an angular frequency of 0.2 to 2%. As shown in Figure 1, the complex viscosity of hydrogels decreases by increasing the angular frequency, representing a shear-thinning property of hydrogels. The hydrogel’s viscosity increases slightly with the increase in the PG content. The hydrogel with 70% *w*/*w* PG content has the highest storage and loss modulus compared to samples with a lower PG content or in the absence of PGs, confirming that PGs improve the viscoelastic properties of hydrogels. All samples showed a higher loss modulus than a storage modulus, indicating that the hydrogel’s viscous property is dominant over elastic properties.

The rheological properties of the collagen suspensions are influenced by the interaction between collagen and PG networks, and the concentration of PGs plays a role in forming this interaction through the entanglements of the network [40]. Due to the heterogeneous composition of the PGs used in this study—including their relative differences in polymer chain length, molecular structure (e.g., linear versus branched), and molecular weight—these ECM components became intricately intertwined with the collagen network, as shown in the SEM images. Hence, the dynamics of the chains are significantly hindered by these entanglements. The addition of PGs leads to the emergence of a nearly constant storage modulus across a wide range of frequencies (Figure 1B). The analysis of the region of constant G’ in the plateau modulus showed minimal relaxation within this frequency range (relaxing back to an equilibrium orientation and configuration following the removal of shear stress), indicating the hindering of molecular relaxation by the entanglements. As the amounts of PGs increase, the range of frequencies over which G’ remains independent of frequency expands, thus reflecting the larger number of entanglements per chain and the more hindered relaxation process [40,41].

### 3.2. FTIR

**Figure 2:**
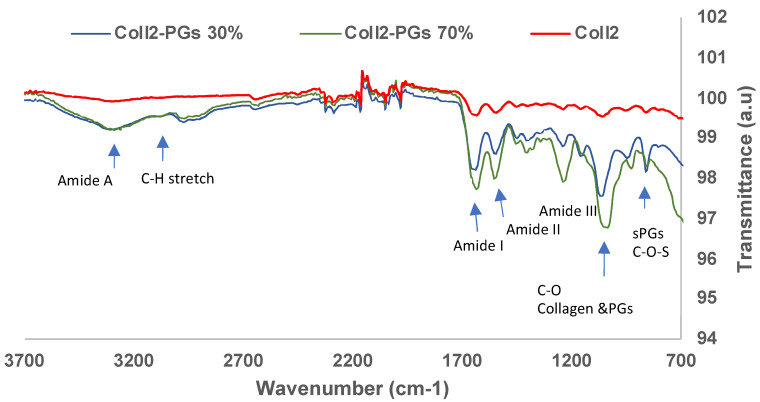
FTIR spectra of simulated nucleus pulposus models with varying concentrations of PGs.

Figure 2 shows the averaged (n = 3) FTIR spectra of collagen II–PGs (0, 30, 70%); the FTIR spectra of all samples showed characteristic collagen amide A, I, II, and III bands. The CH_2_ side chain vibration peak at 1338 cm^−1^ is also a key characteristic feature of collagen, which was present in all samples [42]. IR spectra of collagen II and PGs have a significant overlap due to the presence of similar functional groups. There are significant changes (<4 cm^−1^) in the frequency of amides I, II, and III between the samples (Table 1). A significant reduction in transmittance occurred by adding PGs to collagen, which is correlated to the quantity of PGs in the samples. The increase in the PG content shifted the amide I and C-O band to a lower wavenumber. The peak intensity (transmittance) of amide I, amide II, and amide III bands for the Coll2-PG 70% hydrogel significantly decreased, suggesting an increase in the amount of the functional groups (C=O stretch, C-N stretch, and N-H bend) due to the presence of a higher amount of PGs. Thus, the shift in the peak positions and the changes in the intensity of the bands for different formulations suggest the likelihood of a bond between collagen and PGs (a hydrogen bond between carboxylic and amide groups).

### 3.3. Cryo-Scanning Electron Microscopy

**Figure 3:**
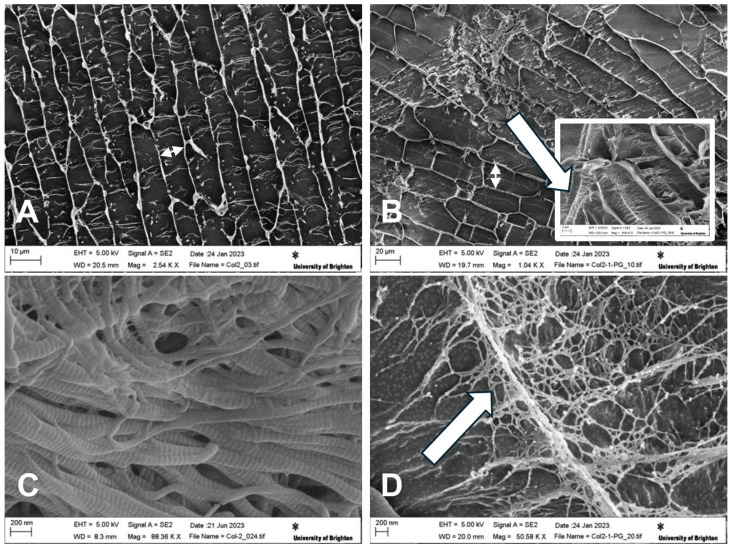
Cryo-SEM micrographs of NP-mimicking constructs. Micrographs show the planar structure of (**A**) collagen II (size bar 50 mm) and (**B**) collagen II–PG 30% (size bar 10 mm). The double-headed arrows highlight the increased distance between collagen sheets, and the insert in (**B**) shows PGs both deposited on and interposing between collagen II fibres (arrow). (**C**) Detailed structure of collagen II showing the typical d-spacing/banding characteristic of collagen fibrils (size bar 200 mm). (**D**) Detailed structure of PGs interposed between collagen II fibres forming thin treads branching towards the collagen fibres (arrow) (size bar 200 mm). Cryo-SEM images were taken at the surface of sublimed samples at 5 KV and different magnifications.

SEM imaging was performed in the cryogenic state to assess the morphological features of the hydrogel core structures. An interconnected planar structure was observed for the hydrogels with and without PGs; fibrous struts were also observed between the collagen sheets (Figure 3A,B). Collagen samples in the absence of PGs have a rather regular pattern of elongated pore structures (Figure 3A). However, it can be observed that collagen fibrillogenesis in the presence of PGs seems to have created a less uniform pore structure compared to pure collagen (Figure 3B). The addition of PGs produced larger pores, with areas of dense PG domains being observed as randomly distributed across the hydrogel. Higher magnification microscopy shows the formation of a secondary network/mesh of PGs with finer struts between the primary network of collagen (Figure 3B, insert). Cryo-SEM confirmed the formation of collagen fibrils with their typical striated d-band structure (Figure 3C), while PG formed thin treads located within the porosity of the collagen type II (Figure 3D). These treads branched out towards the collagen type II fibrils, coating them (Figure 3B, insert) and leading to the reduction in their diameter from 129.08 ± 17.3 nm to 53.2 ± 4 nm (measured using ImageJ, n = 15 per group).

### 3.4. Water Retention Capacity

**Figure 4:**
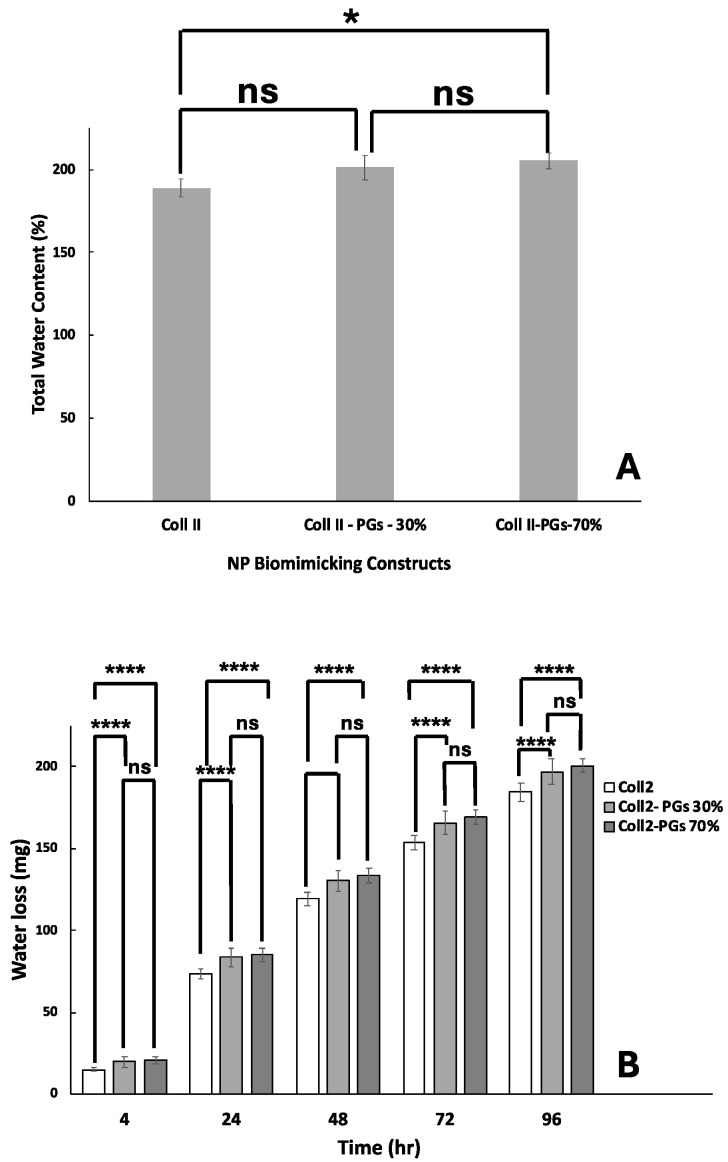
Water content and retention in biomimicking nucleus pulposus constructs. (**A**) Total water content. (**B**) Water loss over a period of 4 days. The symbol ‘ns’ indicates a *p*-value > 0.05, and ‘*’ and ‘**** ’ indicate *p*-values of <0.05 and <0.0001, respectively.

As shown in Figure 4A, the hydrogel’s initial water content (having the same volume of 200 µL) was increased as the PG ratio increased. PGs are highly hydrophilic, and their addition to collagen gel could give rise to a higher water retention capacity. The water loss of pure collagen II samples is significantly less than samples containing PGs (*p* < 0.0001). However, there is no significant difference between the water loss of samples with 30 and 70% PGs. Although the introduction of PGs results in the higher initial water retention capacity of the hydrogel, it also gives rise to the formation of a higher porous structure and interconnectivity with larger pore dimensions according to Cryo-SEM images; hence, the introduction of a higher amount of PGs increased the water evaporation/loss (Figure 4B).

### 3.5. Cellular Characterization

**Figure 5:**
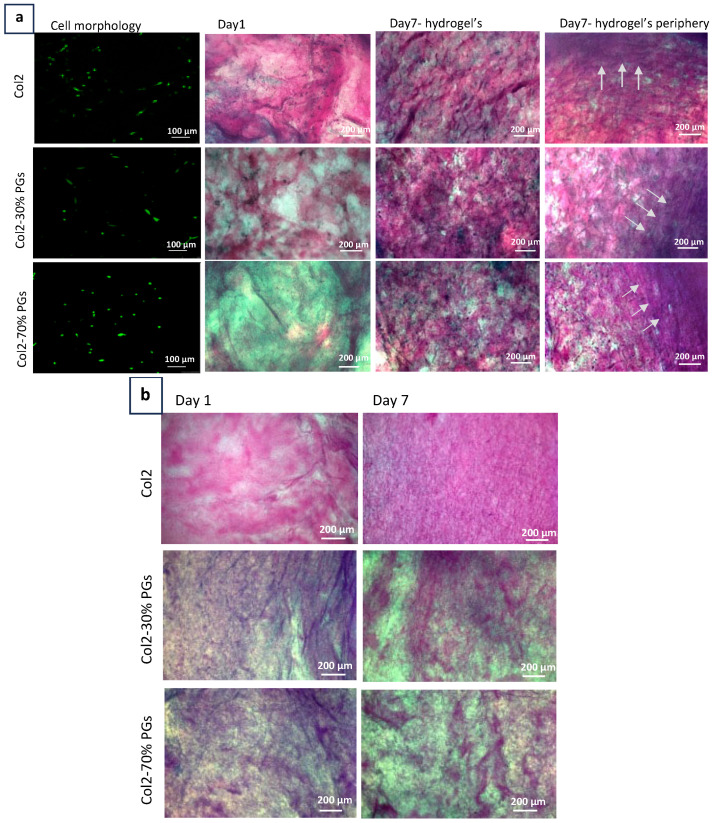
Cell and ECM characterization. (**a**) Cell morphology and viability (green) and histological evaluation of NP-mimicking hydrogels. Collagen was detected by pink staining with picrosirius red, and PGs were detected by blue staining obtained with Alcian blue. White arrows indicates the formation of a more compact ECM and radial organization of collagen fibres moving from the centre to the periphery of the NP constructs was observed, particularly in the case of constructs including 70% proteoglycans. (**b**) Acellular construct staining with picrosirius red and Alcian blue; the radial orientation of collagen fibres is not observed in these constructs.

Figure 5a depicts the morphological changes and the synthesis of extracellular matrix components in different hydrogel compositions. Cells showed an elongated morphology with evidence of mitosis in the absence of PGs, while they adopted a more rounded morphology in the presence of PGs. These observations are aligned with the degeneration process, where mature human NP cells undergo phenotypic changes and shift from a chondrocyte-like round morphology to an elongated fibroblast-like morphology.

Higher amounts of collagen and PGs appeared to be synthesized by NP cells at increasing PG content. This indicates that the increased PG content of hydrogels creates a more favourable environment for the NP cells to synthesize and secrete these extracellular matrix components. The reduction in collagen and PG synthesis in response to lower PG content gives rise to reduced hydrogel structural integrity and functional properties. Notably, when moving from the centre to the periphery of the constructs, NP cells produced a denser ECM with radially organized collagen fibres. This fibre orientation was not observed in acellular constructs (Figure 5b). The presence of randomly organized fibres at the centre and the transition to more organized fibres as we move toward the periphery mimics the natural tissue, where there is no distinct boundary between the NP and AF [44]. This gradual structural change ensures a graded transition between NP and AF.

**Figure 6:**
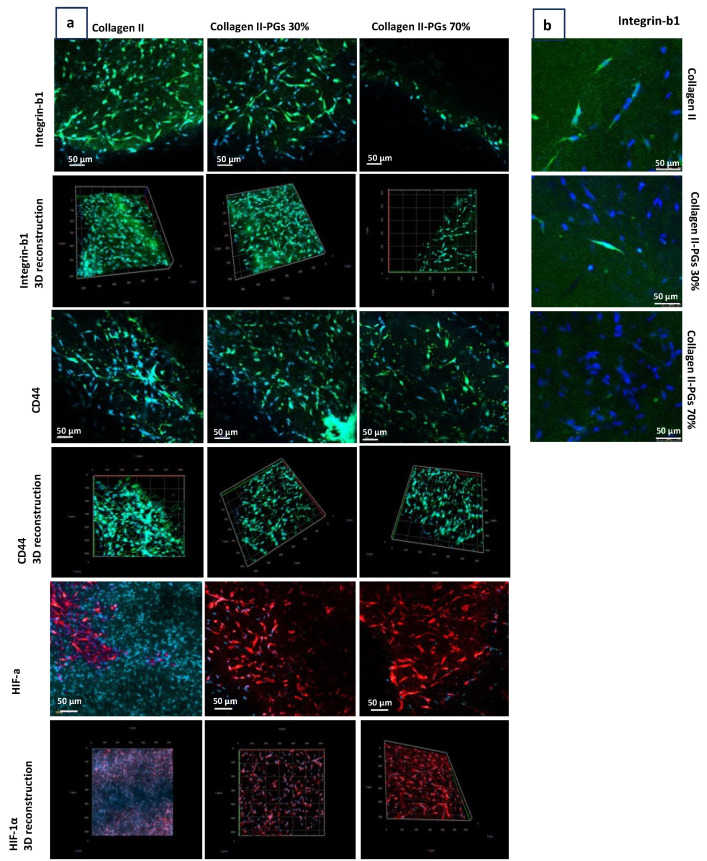
Confocal microscopy of NP cells encapsulated in ECM constructs. (**a**) Integrin-β1, CD44, and hypoxia-inducible factor-1α (HIF-1α) immunohistochemistry staining images obtained with a 10× objective lens. (**b**) Integrin-β1 imaging obtained with a 20× objective lens. The effect of PGs on key biomarker expression. Three-dimensional reconstructions generated from z-stack confocal imaging (20 μm depth) showing the spatial distribution of integrin-β1, CD44, and HIF-1α within the hydrogel.

Immunohistochemistry analysis confirmed integrin-β1-receptor-mediated adhesion of the cells to the collagen type II fibres in the absence or at lower PG amounts, where the expression of the CD44 hyaluronic acid receptor increased (Figure 6). Increased PG content of the substrate results in a denser and more hydrated matrix, which can create a physical barrier between the integrin-β1 receptor on the cell surface and the collagen fibres. In addition, the higher concentration of PGs in the substrate can lead to steric hindrance, limiting the accessibility of the integrin-β1 receptor to collagen fibres. These findings suggest that PGs can modulate the cell–matrix signalling pathway by the activation of CD44 receptors. This modulation can have significant implications for cell adhesion, migration, cytoskeletal organization, and gene expression, ultimately affecting the behaviour and function of NP cells within IVD. These findings also indicate that the increased PG content leads to the higher expression of hypoxia-inducible factor (HIF-1a) by NP cells. This suggests a potential regulatory role of PGs in HIF signalling. The effect of PGs on the expression of HIF-1a by NP cells is not established in the existing research. PGs, through their interaction with the ECM, can affect the diffusion of oxygen within the substrate; an increased PG content may influence the oxygen tension experienced by the NP cells. A decrease in oxygen tension due to altered substrate composition may lead to increased HIF-1a expression. Furthermore, PGs can interact with various signalling pathways, including growth factor signalling and inflammation. The increased PG content may modulate these signalling pathways in a way that promotes HIF-1α expression. It is important to note that the role of HIF-1a in the regulation and progression of IDD is complex and depends on the oxygen tension within the microenvironment of IVD. In moderate hypoxia, HIF-dependent pathways play a beneficial role. However, in severe hypoxia, these pathways can have detrimental effects, turning HIF-1a into a double-edged sword in the context of IDD.

### 3.6. Discussion

The physicochemical properties of the substrate, obtained without the use of cross-linkers, were found to be key in explaining the observed cellular responses as they correlated with variations in PG content within the constructs. Specifically, a reduction in the viscoelastic properties of collagen–PG dispersions at decreasing PG concentrations was observed. Noticeably, the gels were obtained through a gelification method of the monomeric collagen at body temperature. At that stage, a minimum level of contraction may take place, but this did not affect the overall size of the samples that remained adherent to the walls of the tissue culture plate well. After this time, the experiments were performed for relatively short periods of time, and no significant degradation was detected. The physical and chemical interactions between collagen and PGs were confirmed by SEM and FTIR, respectively. These analyses elucidate changes in the rheological behaviour of the hydrogels, where increased PG concentrations promote greater entanglements within collagen and PG networks, resulting in a notable enhancement of both the elastic and viscous modulus. These rheological alterations significantly influenced the microstructure of the constructs; higher PG concentrations resulted in a less organized structure resembling the native, randomly organized healthy NP tissue. In a tridimensional electron tomography study on the bovine cornea, Lewis et al. (2010) showed the intersection of perpendicular proteoglycans between collagen fibres [45]. However, this technique did not provide high-magnification details of the interactions, and the study also did not investigate the effect of different ratios on extracellular matrix structuring. In the present work, SEM images not only confirmed the perpendicular interposition of PG between collagen fibres, but also unveiled their mesh-like structure and their almost periodic points of contact with the collagen fibres and provided clear indications that PG loss due to IVD degeneration leads to more fibrotic-like tissue characterized by more tightly entangled and aligned collagen sheets.

Furthermore, these findings highlighted the importance of water content within the constructs, as higher PG concentrations corresponded to increased water content. This observation aligns with the existing knowledge that NP water loss plays a pivotal role in IVD degeneration, contributing to a decline in mechanical tissue properties [46,47]. In this study, proteoglycans derived from bovine nasal cartilage were used; these are primarily composed of aggrecan with preserved chondroitin sulfate and keratan sulfate side chains. While these commercially available PGs offer consistency and ease of use, their glycosaminoglycan chain lengths and sulfation patterns may differ from those of native aggrecan extracted from nucleus pulposus (NP) tissue. These molecular differences could influence the water-binding capacity, but they are unlikely to have affected the study of cell phenotype observed in this study. As cyclic compression plays a key role in fluid exudation from degenerating discs that may significantly influence the observed water loss behaviour, future investigations should also incorporate dynamic testing protocols, including broader frequency sweeps at physiological temperatures and cyclic loading regimes mimicking the differences in mechanical stresses periodically occurring because of daily activities and during overnight rest.

The physicochemical characterization of the hydrogel provided critical insights into cell behaviour during degeneration. While in vitro models specifically using collagen-based scaffolds or hydrogels to study intervertebral disc (IVD) degeneration are limited, some studies have utilized collagen hydrogels to investigate IVD-related cellular responses. For instance, Hwang et al. employed a microfluidic device containing a collagen hydrogel to examine interactions between IVD cells and endothelial cells under inflammatory conditions [10]. Additionally, collagen-based hydrogels have been explored for their potential in IVD regeneration. Hydrogels composed of type II collagen and hyaluronic acid, chondroitin sulfate, collagen–alginate, and collagen–gelatin have been developed for NP tissue engineering [48,49,50,51,52,53]. While these studies focus primarily on regenerative approaches, they provide valuable insights into the interactions between collagen-based scaffolds and IVD cells, which could be relevant for developing in vitro models of IVD degeneration. Building upon these approaches, our research integrates monomeric collagen type I, collagen type II, and proteoglycans—the main constituents of NP ECM—providing a more advanced biomimetic model compared to most previous studies. This model not only better mimics the native ECM microenvironment but also allows for a more precise investigation of how ECM compositional changes drive cellular responses in IVD degeneration.

We observed that the microstructure of the construct influenced NP cell phenotype, with higher PG contents promoting a round cell shape phenotype [54], whereas lower PG contents favoured an elongated fibroblastic phenotype. These shifts in cell phenotype, driven by substrate composition and structure, exerted an influence on cell phenotype as well as ECM structural component deposition [10,47]. As shown by histological analysis, the decrease in PG concentration within the construct led to a marked decrease in the production of collagen after 7 days.

Notably, the results indicated that NP cell adhesion to the substrate exhibited variations under degenerative conditions, with a reduced PG concentration favouring integrin-β1-mediated adhesion, while a higher PG concentration was associated with CD44-mediated adhesion, which is characteristic of a healthy NP with elevated PG content [55]. Additionally, the study proved that a higher PG concentration correlated with increased expression of the HIF-1α biomarker, consistent with the hypoxic environment typically found in healthy IVD [47,55]. This is likely to be caused by the entrapment of the cells in the proteoglycan-rich matrix, limiting the diffusion of oxygen. This interpretation is corroborated by the reduction in HIF-1a expression in cells colonizing the wider mesh of collagen hydrogels as observed by SEM. Molecular biology techniques such as Western blotting, flow cytometry, and RT-qPCR would provide valuable confirmation of phenotypic shifts and ECM-related gene expression. However, there are significant technical challenges in extracting cells/cell lysate and proteins from cells encapsulated within the collagen- and proteoglycan-based hydrogel matrix, which we found could compromise the reliability and reproducibility of such data.

Here, we support the idea that the identification of the proteoglycan content threshold at which cells enter pathological phenotypical switches can provide new elements of diagnosis and early treatments of degenerating intervertebral discs. The demonstrated ability of quantitative MRI to assess the water content in the IVD [56] can be linked to the proteoglycan content to support the clinical assessment of patients and determine early intervention. However, although the data presented here can provide a framework for correlating PG content with biophysical cues, in vivo validation is essential before such thresholds can reliably guide patient-specific diagnostics or therapeutic decisions. Recent advances in quantitative MRI—such as gagCEST, T1ρ, and dGEMRIC—have shown promise in detecting glycosaminoglycan concentrations and mapping early disc degeneration [57,58,59,60].

Any future therapeutic approaches based on the in situ injection(s) of proteoglycans or cell therapy/tissue engineering procedures will thus be fine-tuned in terms of dosage and construct design [60].

## 4. Conclusions

In summary, the present study sheds light on the intricate interplay between ECM composition, substrate physicochemical properties, and NP cell activity in the context of degeneration whereby any age-related reduction in proteoglycans in the IVD or its invasion by collagen type I following trauma can trigger a pathological loop leading the cells to produce more collagen than proteoglycans. These findings hold promise for advancing the understanding of IVD degeneration and inform the potential development of novel biomaterial-based therapeutics that are able to re-establish healthy IVD ECM conditions.

## Figures and Tables

**Table 1:** FTIR band assignment for collagen II, collagen II–PGs 30%, and collagen II–PGs 70% [43].

Band Assignment	Collagen II	Collagen II–PGs 30%	Collagen II–PGs 70%
**Amide A**	3284	3291	3291
**Amide I (C=O stretch)**	1630	1615	1628
**Amide II (C-N stretch, N-H bend)**	1545	1525	1550
**Amide III, combined with CH_2_ wagging from glycin backbone and proline side chain (C-N stretch, N-H bend, C-C stretch)**	1237	1197	1234
**CH_3_ asymmetric bending**	1451	1413	1417
**COO (stretch of amino side chain)**	1401	1404	1406
**CH_2_ (side chain stretching, characteristic of collagen)**	1334	1305	1342
**C–O–S asymmetric stretching in sulfated PGs**	857	860	800
**C–O stretching of carbohydrate residues in collagen and PGs/SO^3−^ stretching**	1060	1015	1039

## Data Availability

The original contributions presented in the study are included in the article, further inquiries can be directed to the corresponding author.
